# Mitochondrial response to nutrient availability and its role in metabolic disease

**DOI:** 10.1002/emmm.201303782

**Published:** 2014-03-12

**Authors:** Arwen W Gao, Carles Cantó, Riekelt H Houtkooper

**Affiliations:** 1 Laboratory Genetic Metabolic Diseases, Academic Medical CenterAmsterdam, The Netherlands; 2 Nestlé Institute of Health SciencesLausanne, Switzerland

**Keywords:** caloric restriction, diabetes, high-fat diet, metabolic disease, mitochondrial dynamics

## Abstract

Metabolic inflexibility is defined as an impaired capacity to switch between different energy substrates and is a hallmark of insulin resistance and type 2 diabetes mellitus (T2DM). Hence, understanding the mechanisms underlying proper metabolic flexibility is key to prevent the development of metabolic disease and physiological deterioration. An important downstream player in the effects of metabolic flexibility is the mitochondrion. The objective of this review was to describe how mitochondrial metabolism adapts to limited nutrient situations or caloric excess by changes in mitochondrial function or biogenesis, as well as to define the mechanisms propelling these changes. Altogether, this should pinpoint key regulatory points by which metabolic flexibility might be ameliorated in situations of metabolic disease.

## Introduction

Mitochondrial dysfunction is at the center of many metabolic disorders, such as obesity and type 2 diabetes mellitus (T2DM) (Andreux *et al*, 2013). It is widely believed that these disorders can be avoided by regular exercise and restricted food intake. However, compliance is poor and the metabolic syndrome epidemic is still spreading. Therefore, pharmacological approaches to target mitochondrial metabolism are being developed with the aim to treat these disorders.

The mitochondrion is an essential organelle in regulating a number of cellular processes, including energy (ATP) generation through nutrient breakdown, autophagy/mitophagy, and apoptosis. However, it is only sparsely described how these processes are influenced by situations of limited nutrient availability, such as fasting and caloric restriction (CR), or by excessive nutrient supply, such as high-fat feeding.

In this review, we aim to integrate the current knowledge on these areas and address three key questions: (i) What is the evidence for mitochondrial dysfunction in acquired metabolic disorders? (ii) How does energy stress impact on mitochondrial function, for instance at the level of biogenesis (AMPK/sirtuins) and mitochondrial dynamics (fusion/fission/mitophagy)? (iii) Is mitochondrial function a realistic target for pharmacological or nutritional intervention for the treatment of metabolic diseases?

## Deficient metabolic flexibility as a cause for metabolic disease

The capacity of an organism, an organ, or a single cell to adapt fuel oxidation to fuel availability is termed metabolic flexibility (Galgani *et al*, 2008). This nomenclature was proposed as the capability to switch from fat metabolism in a fasting state to suppression of fat oxidation and enhanced glucose metabolism upon stimulation by insulin (Kelley & Mandarino, 2000). Active storage of nutrients and efficient disposal of blood glucose after a meal are characteristic of healthy metabolism (Storlien *et al*, 2004). In turn, metabolically inflexible individuals fail to adapt their fuel preference to changes in carbohydrate or lipid supplies (Galgani *et al*, 2008).

Imbalanced energy homeostasis is a characteristic of obese and type 2 diabetes mellitus (T2DM) patients. Interestingly, metabolic inflexibility can already be observed in pre-diabetic individuals suffering from insulin resistance (Corpeleijn *et al*, 2009), limiting the ability to switch from fatty acid breakdown to fat storage after a meal and vice versa (Kelley, 2005). Diverse mitochondrial parameters vary between insulin-resistant and insulin-sensitive subjects, such as mitochondrial number, structure, and function. This suggests that mitochondrial dysfunction might contribute to metabolic inflexibility and insulin resistance (Galgani *et al*, 2008).

Mitochondrial deficiencies limit the capacity of oxidative tissues to adapt fat oxidation to fat availability, leading to the accumulation of lipids in non-adipose tissues, such as skeletal muscle. Intramyocellular lipids (IMCL) are used as fuel during regular exercise (Coen & Goodpaster, 2012), but lipid accumulation in non-adipose tissues is also highly associated with insulin resistance (Canto & Auwerx, 2009; Corpeleijn *et al*, 2009; Schooneman *et al*, 2013). For instance, IMCL levels were increased in first-level relatives of T2DM patients and associated with impaired insulin-stimulated glucose uptake in muscle (Pan *et al*, 1997). The link between IMCL and insulin resistance was confirmed in biopsies from individuals with obesity and T2DM (Goodpaster *et al*, 2000; Coen & Goodpaster, 2012). In obese individuals, impaired mitochondrial function might contribute to lower IMCL turnover and lead to lipotoxicity (Schooneman *et al*, 2013). These lipids interfere with insulin signaling and represent one of the key aspects that could contribute to insulin resistance in situations of high-fat feeding or lipid infusion (Pan *et al*, 1997; Krssak *et al*, 1999; Goodpaster *et al*, 2000).

Gene-clustering approaches supported the above hypothesis by demonstrating that muscles from T2DM patients display downregulation of lipid oxidation and mitochondrial metabolism genes (Mootha *et al*, 2003; Patti *et al*, 2003). Mitochondrial deficiencies were also characteristic in age-related insulin resistance (Petersen *et al*, 2003). In a parallel study, insulin resistance in the skeletal muscle of insulin-resistant offspring of T2DM patients was associated with dysregulation of intramyocellular fatty acid metabolism, possibly due to deficiencies in mitochondrial oxidative phosphorylation capacity (Petersen *et al*, 2004). In light of the above data, increasing mitochondrial content could constitute a promising preventive and therapeutic approach against insulin resistance and T2DM (Canto & Auwerx, 2009). Several pharmacological and nutritional interventions have been designed in this direction, which protect against the development of insulin resistance and high-fat diet (HFD)-induced metabolic damage (e.g., resveratrol or PPARβ agonists) (Andreux *et al*, 2013).

Although the correlative evidence around mitochondrial dysfunction and the development of metabolic diseases is overwhelming, some evidence casts a shadow of doubt on its causal role. For instance, some of the mouse models that overexpress proteins promoting mitochondrial biogenesis display a largely enhanced mitochondrial content in skeletal muscle, but are insulin resistant (Finck *et al*, 2005; Choi *et al*, 2008). Along the same line, insulin-stimulated glucose sensitivity is increased in some mouse models of mitochondrial dysfunction (Wredenberg *et al*, 2006; Pospisilik *et al*, 2007; Zechner *et al*, 2010). Also, mitochondrial dysfunction is observed only after prolonged high-fat feeding, while insulin resistance develops earlier (Hancock *et al*, 2008). Finally, T2DM patients, despite having a 30% lower mitochondrial content, have higher mitochondrial oxidation rates and do not have an intrinsic problem to increase their whole-body fat oxidation rate during exercise (Larsen *et al*, 2009; Holloszy, 2013). Similarly, the decreased mitochondrial content in insulin-resistant old individuals seems to be rather linked to decreased physical activity and can be perfectly enhanced in response to exercise (Broskey *et al*, 2014). This highlights how some tissues, such as human muscle, might actually have a notable reserve of respiratory capacity, and that minor changes in their mitochondrial content might not be enough to limit resting respiratory needs. Indeed, the concept of reserve respiratory capacity in humans is not new. It was initially characterized in diverse human cell lines, where COX activity was found to be higher than that required to support endogenous respiration rates, allowing this way some adaptability to higher respiratory needs (Villani *et al*, 1998). Therefore, while many strategies to increase mitochondrial function rendered protection against insulin resistance and T2DM, further studies are required to clarify the link between mitochondrial deficiency and insulin resistance. Furthermore, future studies will have to address mitochondrial functional quality rather than quantity, as will become apparent in the following sections.

## Mechanisms controlling mitochondrial metabolism

In order to maintain metabolic homeostasis, organisms adjust the capacity and efficiency of ATP generation to changes in energetic demand and supply. Mitochondrial activity can be controlled through at least two major mechanisms: acute ones, aimed to qualitatively modify mitochondrial function, and longer-term transcriptional mechanisms, aimed to increase mitochondrial number.

### Acute mechanisms for the control of mitochondrial metabolism

Acute mechanisms to modify mitochondrial function affect the intrinsic ability of mitochondria to generate ATP per molecule of nutrient or per unit of time. Two major mechanisms have potently emerged, namely changes in mitochondrial architecture or dynamics, and regulation through post-translational mechanisms.

#### Mitochondrial dynamics

**Figure 1:**
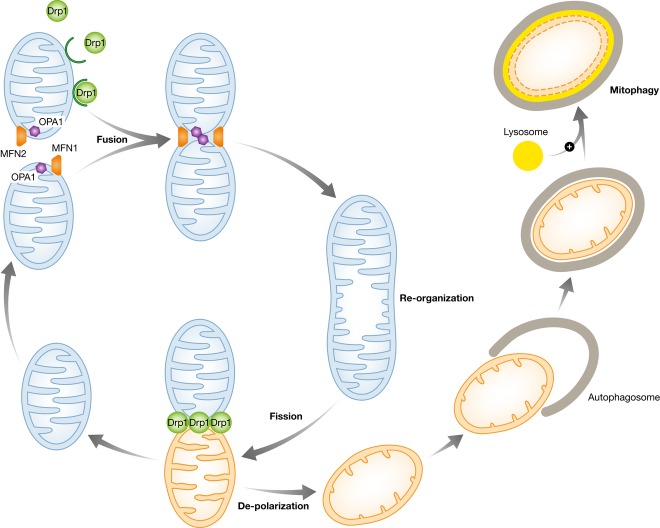
Mitochondrial dynamics and quality control. Mitochondrial dynamics involves repetitive cycles of mitochondrial fusion and fission. Fusion of mitochondria is regulated by mitofusin (Mfn) 1 and Mfn2 (both proteins are required for mitochondrial outer membrane fusion) and optic atrophy 1 (Opa1) (required for mitochondrial inner membrane fusion protein). Mitochondrial fission is regulated by dynamin-related protein 1 (Drp1), mitochondrial fission factor (Mff), and fission 1 (Fis1). When a daughter mitochondrion is dysfunctional/depolarized, it will be targeted for elimination. The defective mitochondria accumulate tensin homolog-induced putative kinase protein 1 (PINK1) at the mitochondrial surface, which in turn recruits Parkin. Parkin-induced ubiquitylation of the outer membrane initiates the recruitment of autophagosomes, which are degraded after fusion with a lysosome.

Changes in mitochondrial architecture are dynamic and involve a delicate balance between proteins promoting fusion [e.g., mitofusin (Mfn) 1 and Mfn2, and optic atrophy 1 (Opa1)] and fission [e.g., fission 1 (Fis1), mitochondrial fission factor (Mff), and dynamin-related protein 1 (Drp1)] (Youle & van der Bliek, 2012). Cells under a well-fed condition maintain their mitochondria in a separated (or fragmented) state, while under fasting or starved conditions, mitochondria tend to persist in a connected (or fused) state (Gomes *et al*, 2011). This suggests that adaption of bioenergetics involves remodeling of mitochondrial architecture. Indeed, as described below, changes in mitochondrial architecture can affect respiratory complex assembly and the proper coupling of respiration to ATP synthesis (Gomes *et al*, 2011). In addition, the transitions between fragmented and fused states allow mitochondria to reorganize and dispose damaged elements through mitophagy (Fig 1). Notably, the mitochondrial life cycle is attenuated when the fusion or fission pathways are not functioning properly, leading to mitochondrial dysfunction. Dysfunctional mitochondria, especially those with dissipated membrane potential, are recognized by the mitophagic machinery and broken down rapidly (Youle & Narendra, 2011). Thus, mitochondrial architectural changes might offer a new molecular mechanism to connect nutrient availability to bioenergetic adaptations (Liesa & Shirihai, 2013).

#### Post-translational modifications

**Figure 2:**
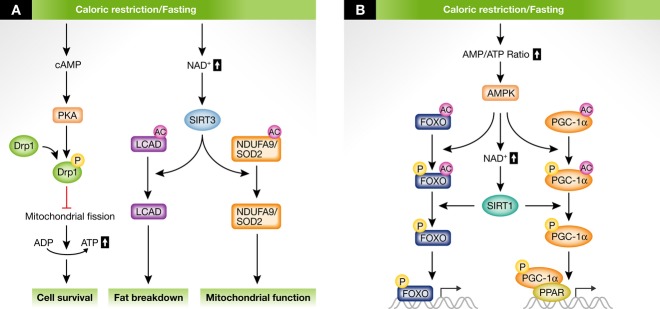
The acute (A) and transcriptional (B) regulation of mitochondrial networks upon caloric restriction and fasting. (A) When nutrients are limited, cAMP levels rise and activate protein kinase A (PKA). PKA in turn phosphorylates and inactivates Drp1, thereby blocking mitochondrial fission. This relative switch to elongated mitochondria increases ATP levels as a response to nutrients scarcity. Nutrient scarcity also increases the level of mitochondrial NAD^+^, leading to the activation of SIRT3 and improving mitochondrial function through deacetylation of ket SIRT3 targets (e.g., mitochondrial complex I protein NDUFA9, the superoxide dismutase 2 (SOD2), and fatty acid oxidation enzyme LCAD. (B) Upon caloric restriction and fasting, AMP-activated protein kinase (AMPK) is activated by an increase in the ratio of AMP relative to ATP. AMPK phosphorylates several transcriptional (co)activators, such as PCG-1α, forkhead box O (FOXO). This primes these regulators for SIRT1-dependent deacetylation, which is further enhanced by a concurrent increase in NAD^+^ levels.

**Figure 3:**
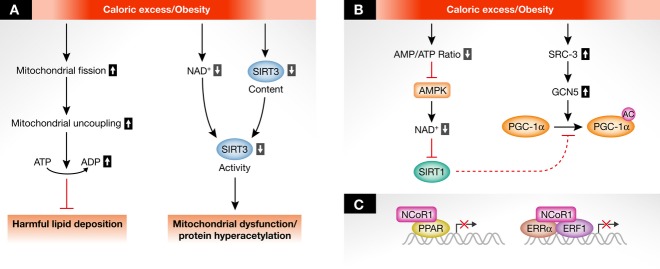
The acute (A) and transcriptional (B, C) regulation of mitochondrial networks upon caloric excess and obesity. (A) Upon caloric excess, lipid overload triggers mitochondrial fission, which is accompanied by mitochondrial uncoupling and ATP depletion. The enhanced mitochondrial uncoupling may be a solution to consume excess energy and prevent fat deposition in the cells. At the same time, decreased NAD^+^ levels and reduced SIRT3 protein content lead to SIRT3 activity, mitochondrial hyperacetylation, and mitochondrial dysfunction. (B) Caloric excess inhibits AMPK activity because of high intracellular ATP levels. This reduces NAD^+^ levels and SIRT1 activity. As a consequence, the transcriptional regulators that are subject to SIRT1 deacetylation are attenuated. This is further emphasized by the activation of steroid receptor coactivator protein 1 (SRC3) and the acetyltransferase GCN5. (C) During high-fat diet feeding, the nuclear receptor corepressor 1 (NCoR1) is upregulated and thereby represses the activity of key transcription factors that modulate mitochondrial activity, such as nuclear respiratory factor 1 (NRF1), NRF2, and estrogen-related receptors (ERRs), as well as peroxisome proliferator-activated receptors (PPARs).

Among different post-translational modifications, protein acetylation has recently emerged as another reversible mechanisms linking the metabolic state and mitochondrial function (Figs 2A and 3A). Mitochondria contain many acetylated proteins, often at several residues. Reversibly acetylated mitochondrial proteins can be found in most mitochondrial functions, including oxidative phosphorylation (Ahn *et al*, 2008), reactive oxygen species metabolism (Qiu *et al*, 2010; Chen *et al*, 2011) and fatty acid metabolism (Hirschey *et al*, 2010). Upon fasting and high-fat feeding, the acetylation pattern of mitochondrial proteins strongly changes. This is due to changes in the balances between acetylation and deacetylation rates, for instance involving the mitochondrial NAD^+^-dependent deacetylase SIRT3 (Houtkooper *et al*, 2012). This opens the possibility that coordinated changes in mitochondrial acetylation serve to regulate mitochondrial acetyl-CoA levels and trigger appropriate responses. Therefore, understanding the specificity of mitochondrial acetylation dynamics will be a key to evaluate the impact of nutritional states on mitochondrial function. In addition to acetylation, novel mitochondrial post-translational modifications have emerged that may reflect the metabolic state, including succinylation and malonylation (Du *et al*, 2011; Peng *et al*, 2011). Although the physiological role of these modifications is not yet understood, its reliance on the NAD^+^-dependent SIRT5 suggests a metabolic function analogous to SIRT3-dependent acetylation.

### Transcriptional regulation of mitochondrial metabolism

Cellular oxidative capacity can also be controlled through increasing its mitochondrial content. Mitochondrial biogenesis is achieved through orchestrated induction of several transcriptional regulators, whose activity is critically determined by energetic demands (Andreux *et al*, 2013) (Figs 2B, 3B and C). A key family of transcription factors that connects lipid metabolism with transcriptional outputs is the PPARs. PPARs are nuclear receptors that directly bind specific lipid species that generally increase upon cellular fat overflow in the cells. There are three PPARs: α, β (also known as δ) and γ. Each of these binds specific lipid species as ligands and controls different transcriptional programs linked to lipid metabolism (Wahli & Michalik, 2012). For example, PPARα activates fatty acid oxidation by inducing lipid oxidation genes in mitochondria and peroxisomes (Yoon, 2009). PPARβ/δ triggers fatty acid β-oxidation in muscle and adipose tissue, which results in enhanced fat breakdown (Wang *et al*, 2003; Varga *et al*, 2011). In contrast, PPARγ controls lipogenic and adipocyte differentiation gene expression and activates lipid storage (Varga *et al*, 2011; Wahli & Michalik, 2012).

The PPARγ coactivator 1α, PGC-1α, regulates mitochondrial biogenesis by coactivating various transcription factors and nuclear receptors. The activity of PGC-1α is regulated by post-translational modifications that fine-tune mitochondrial metabolism with environmental challenges (Canto & Auwerx, 2009). For example, PGC-1α activity is determined by its acetylation status. In the basal state, PGC-1α is acetylated, which dampens PGC-1α activity. Upon nutrient stress, the NAD^+^-dependent deacetylase SIRT1 activates PGC-1α, a reaction that is rate-limited by availability of the metabolite NAD^+^ (Houtkooper *et al*, 2010a). Hence, increasing NAD^+^ content leads to higher SIRT1 activity (Canto & Auwerx, 2012). NAD^+^ is a highly dynamic molecule whose cellular abundance depends on the metabolic state. Therefore, SIRT1 is optimally positioned to translate the metabolic state to transcriptional outputs on mitochondrial biogenesis through the deacetylation of PGC-1α. Indeed, different situations of energy restriction, including fasting and exercise, increase NAD^+^ levels and trigger SIRT1/PGC-1α-dependent mitochondrial adaptation (Lin *et al*, 2000; Houtkooper *et al*, 2012). In addition to PGC-1α, SIRT1 also deacetylates forkhead box O (FOXO) transcription factors, enhancing the expression of lipid oxidation and oxidative stress protection genes (Brunet *et al*, 2004). Interestingly, FOXOs can also act as critical regulators of mitochondrial homeostasis through, at least, two additional means: (i) by regulating the expression of Mul1, a mitochondrial E3 ubiquitin ligase that controls Mfn2 degradation rates (Lokireddy *et al*, 2012), and (ii) by regulating the expression of the PTEN-induced kinase 1 (PINK1), a key component of the mitophagy machinery (Mei *et al*, 2009). This intricate transcriptional network constitutes an excellent mechanism to fine-tune oxidative metabolism and longer-term adaptations.

### Regulation of mitochondrial metabolism upon nutrient scarcity

#### Acute regulation

Nutrient scarcity in organisms is linked to an increase in circulating glucagon and other stress hormones, such as norepinephrine, which generally increase intracellular cAMP levels and PKA signaling (Fig 2A). Under these circumstances, mitochondrial fusion is typically observed (Liesa & Shirihai, 2013) (Fig 2A). Elongated mitochondria upon fasting possess more cristae and increased levels of dimerization and activity of ATP synthase, ensuring a better coupling of fuel oxidation with ATP synthesis (Gomes *et al*, 2011). These changes in mitochondrial cristae content could also contribute to an optimal assembly of so-called respiratory supercomplexes, promoting a more efficient electron flux through the respiratory chain (Cogliati *et al*, 2013). On the other hand, mitochondria that fail to elongate are degraded by mitophagy. But, how does this happen at the molecular level? Mitochondrial fusion upon energy stress requires the mitochondrial oligomerization of Drp1, which actively shuttles between mitochondria and the cytosol. The activation of cAMP/PKA upon nutrient starvation leads to phosphorylation of Drp1, preventing its stable docking on the mitochondrial surface and therefore mitochondrial fission (Cereghetti *et al*, 2008) (Fig 2A). While this is the most solidly described mechanism for the acute regulation of mitochondrial architecture upon energy deprivation, there might be additional layers of regulation, as reviewed recently (Escobar-Henriques & Anton, 2013; Otera *et al*, 2013), even though their participation on metabolic adaptations has been poorly explored.

The SIRT3 deacetylase enzyme has also emerged as a key regulator of mitochondrial activity (Lombard *et al*, 2007). Upon fasting, SIRT3 expression is rapidly induced and deacetylates key residues on mitochondrial proteins (Hirschey *et al*, 2010). The increased SIRT3 activity facilitates optimal fatty acid oxidation, mitochondrial electron transfer and protection against reactive oxygen special all at once (He *et al*, 2012). Interestingly, SIRT3 also deacetylates and elevates the GTPase activity of the inner membrane fusion protein OPA1, thereby favoring mitochondrial elongation (Samant *et al*, 2014). It is not clear yet whether the increase in SIRT3 activity upon nutrient deprivation is regulated exclusively at the transcriptional level. Being NAD^+^ dependent, it seems likely that SIRT3 is controlled by mitochondrial NAD^+^ levels. Indeed, exogenously providing NAD^+^ precursors can enhance SIRT3 activity in mammalian cells and tissues (Canto *et al*, 2012) and rescue deficient SIRT3 activity in a model for complex I deficiency (Karamanlidis *et al*, 2013). This indicates that fluctuations in NAD^+^ levels during nutrient restriction might contribute in the control of SIRT3 activity. Intriguingly, CR enhances mitochondrial protein acetylation in diverse tissues, such as liver, heart, and kidney (Schwer *et al*, 2009). This may be the result of fat overflow into these tissues. Indeed, the global increase in protein acetylation does not reflect how specific residues are deacetylated upon CR (Hebert *et al*, 2013). This suggests that SIRT3 regulates more accessible hydrophilic residues, while residues on hydrophobic regions are less accessible and prone to become acetylated.

#### Transcriptional responses

Prolonged nutrient stress requires adaptation through transcriptional responses. A primordial sensor for energy stress is the AMP-activated protein kinase (AMPK), an enzyme that is activated by increases in the AMP/ATP ratio (Fig 2B). Upon activation, AMPK activates catabolic pathways and switches off energy-consuming processes. One of these targets is PGC-1α (Jager *et al*, 2007). Upon phosphorylation, PGC-1α is recognized as a deacetylation target by SIRT1 (Canto & Auwerx, 2009; Iwabu *et al*, 2010). As a consequence, PGC-1α coactivates a set of transcription factors involved in mitochondrial metabolism, including estrogen-related receptors, nuclear respiratory factors, and PPARs (Andreux *et al*, 2013). This two-step activation of PGC-1α might be a conserved mechanism for AMPK to confer specificity among the various targets of SIRT1 (Canto & Auwerx, 2010). Other effectors of AMPK are the FOXO transcription factors (Greer *et al*, 2007), which are also a target for deacetylation by SIRT1 (Brunet *et al*, 2004). The deacetylated FOXO will then contribute to the regulation of mitochondrial function and lipid metabolism (Canto & Auwerx, 2009). A second connection between fasting-induced transcriptional adaptation and mitochondrial dynamics lies in the transcriptional regulation exerted by fasting-related hormones. So far, however, this has been a largely unexplored territory. For example, dexamethasone induces Drp1 expression in liver cells and thereby changes mitochondrial morphology and metabolism that favor gluconeogenic procedures (Hernandez-Alvarez *et al*, 2013). To what extent these pathways are also active in other tissues and in other physiological conditions needs to be determined.

Additional interest on how nutrient scarcity modulates mitochondrial function comes from the fact that CR is the most consistent physiological intervention to improve health and extend lifespan across a wide range of organisms (Fontana *et al*, 2010; Houtkooper *et al*, 2010b). The relationship between CR and mitochondrial regulation is, however, still under debate. Initial evidence indicated that CR enhances mitochondrial content (Nisoli *et al*, 2005). These findings were rapidly accepted and have led to the concept that CR induces mitochondrial biogenesis, even though some studies found no increase in mitochondrial protein content upon CR (Hancock *et al*, 2008). Interestingly, a recent report indicates that CR enhances mitochondrial function without changes in mitochondrial content or biogenesis (Lanza *et al*, 2012). The mechanisms underlying this enhanced function are, however, not clear. A conciliatory hypothesis would be that CR protects against mitochondrial dysfunction by improving mitochondrial dynamics and the efficiency of mitophagic processes.

### The role of mitochondria in caloric excess

#### Acute regulation

Lipid overloading promotes fast and transient changes in mitochondrial architecture. Studies in Zucker rats and obese humans have shown that Mfn2 was strongly decreased in their skeletal muscle tissues compared to the control groups, which was associated with smaller mitochondrial network and size (Bach *et al*, 2003). Along with this fusion deficiency, mitochondrial fission is induced within minutes after a lipid overload in INS-1 cells (Molina *et al*, 2009; Liesa & Shirihai, 2013) (Fig 3A). Additional recent efforts have elegantly demonstrated that lipid overload also enhances norepinephrine-induced mitochondrial fission in brown adipocytes *in vivo* and *ex vivo* (Wikstrom *et al*, 2014). These mitochondrial fission events promoted mitochondrial uncoupled respiration (Liesa & Shirihai, 2013; Wikstrom *et al*, 2014). A key issue that requires further study is the basic mechanism by which fission enhances mitochondrial uncoupling. In this sense, evaluating whether fission alters cristae structure and supercomplex assembly will be interesting in the future. Irrespective of the mechanism, mitochondrial uncoupling upon lipid loading may be a way to dissipate energy and prevent detrimental lipid deposition in the cell (Liesa & Shirihai, 2013). In line with the above observations, obese patients display a largely fragmented mitochondrial network in skeletal muscle (Bach *et al*, 2003). The analysis of mice with mitochondrial fusion or fission defects will be key to evaluate the relevance of fusion/fission in the acute adaptation to nutrient challenges. Considering that global deletion of key mitochondrial dynamics proteins, such as Mfn1, Mfn2, Opa1 or Drp1, leads to embryonic lethality (Chen *et al*, 2003; Davies *et al*, 2007; Ishihara *et al*, 2009), tissue-specific knockout models are currently being explored. Indeed, deletion of Mfn2 in the liver disrupts mitochondria-ER connectivity and thereby induces ER stress-mediated susceptibility to metabolic disease upon high-fat feeding (Sebastian *et al*, 2012), indicating once more that the regulation of mitochondrial dynamics is essential to meet metabolic challenges. Defective mitochondrial dynamics and mitochondrial-ER communication in hypothalamic POMC and AgRP neurons might actually modify feeding behavior and whole-body energy homeostasis (Dietrich *et al*, 2013; Schneeberger *et al*, 2013).

Dietary excess of lipids also dramatically changes mitochondrial protein acetylation. One week of HFD enhanced SIRT3 levels and prevented mitochondrial hyperacetylation despite the higher levels of lipid substrates (Hirschey *et al*, 2010). This increase in SIRT3 may represent a cellular adaptation to cope with the need to use fatty acid oxidation as the main path for energy production. Conversely, prolonged HFD dramatically decreases SIRT3 expression, correlating with the appearance of mitochondrial hyperacetylation and mitochondrial dysfunction (Hirschey *et al*, 2010). To date, it is still unclear why the initial adaptations tend to disappear with time, ultimately driving the organism into metabolic disease.

#### Transcriptional responses

Changes in mitochondrial dynamics are apparent in the acute phase of caloric excess. Still, however, Mfn2 expression is also reduced, possibly through transcriptional regulation. Similar to many mitochondrial genes, Mfn2 expression is controlled by PGC-1α (Soriano *et al*, 2006), suggesting that decreased PGC-1α activity explains the defects in Mfn2 observed in obese and T2DM individuals. Indeed, HFD in mice leads to hyperacetylated, inactive, PGC-1α (Coste *et al*, 2008). This is likely regulated at several levels. During HFD, SIRT1 expression is reduced, while the GCN5 acetyltransferase, which acetylates PGC-1α, is expressed at higher levels (Coste *et al*, 2008). HFD also reduces the NAD^+^ content in diverse tissues, which is likely to impair SIRT1 activation (Kim *et al*, 2011; Yoshino *et al*, 2011) (Fig 3B). The resulting hyperacetylation of PGC-1α renders it unable to properly coactivate target transcription factors, leading to decreased mitochondrial biogenesis.

The expression of repressive transcriptional regulators, such as the nuclear receptor corepressor 1 (NCoR1), is also enhanced by HFD, repressing genes that control mitochondrial activity and contributing to the excessive calorie storage (Yamamoto *et al*, 2011) (Fig 3C). In fact, PGC-1α and NCoR1 oppositely regulate several transcription factors, including nuclear respiratory factors, estrogen-related receptors and PPARs (Scarpulla, 2011; Andreux *et al*, 2013; Mottis *et al*, 2013). An interesting question is why mitochondria can perfectly respond to acute caloric excess, but fail to do so when the intervention persists for a long time. In the initial phases of HFD (up to 1 month), mitochondrial and lipid oxidation genes are upregulated to meet the higher flux of lipid substrates (Garcia-Roves *et al*, 2007; Hancock *et al*, 2008). However, prolonged lipid overload compromises mitochondrial function. The threshold for this to happen and the reason behind it still need to be solved, but it seems likely that this defective long-term protection contributes to lipid accumulation and insulin resistance.

## Discussion

Revealing the causal link between mitochondrial function and metabolic disorders continues to be a great challenge. Based on the evidence during the last decade, the concept that T2DM and obesity are linked to mitochondrial dysfunction has gathered momentum in the scientific community. However, most of the data supporting this concept are based on the fact that reductions in the content of mitochondrial enzymes and alterations in mitochondrial shape were observed in the muscles from patients with T2DM. This went hand in hand with the accumulation of lipids that could impair insulin signaling. It was therefore hypothesized that increasing mitochondrial function might be a valuable strategy to boost lipid oxidation, prevent lipid accumulation, and thereby treat insulin resistance. However, the above-mentioned hypothesis has three conflicting points: (i) mitochondrial biogenesis can occur simultaneously to insulin resistance, (ii) disruption of mitochondrial function leads to increases in basal and insulin-stimulated glucose uptake in some models, and (iii) most studies actually show that fat oxidation is increased in obese, insulin-resistant individuals and T2DM patients.

While most studies observe mitochondria in a steady state, mitochondrial function can dramatically change through fusion/fission or post-translational modifications of mitochondrial proteins. We propose that metabolic flexibility and mitochondrial fitness might not necessarily correlate with mitochondrial number or mitochondrial protein content, but to the ability to adapt mitochondrial activity and biogenesis to external stimuli. Indeed, higher mitochondrial content could also be due to deficiencies in mitochondrial recycling mechanisms and in fact be linked to mitochondrial dysfunction. This is the case, for example, in muscle-specific AMPK knockouts, which display enlarged but dysfunctional mitochondria (O'Neill *et al*, 2011).

In light of the evidence reviewed here, the evaluation of mitochondrial content as a readout for function might lead to conflictive observations on the relationship between diverse pathologies, lipid oxidation rates, and true mitochondrial function. This advocates for the need to determine various additional aspects of mitochondrial well-being to certify changes in mitochondrial function within pathophysiological states. Still, some key questions remain unanswered, such as (i) why does mitochondrial content decline upon a certain length of fat overfeeding and how can this be prevented, and (ii) can strategies aimed to enhance insulin sensitivity be successful without proper mitochondrial fitness? Needless to say, interesting times lie ahead.

## Pending issues%

How does mitochondrial dynamics influence metabolic activity and quality control in response to physiological cues?

Solidify the contribution of mitochondrial dynamics in the development of human metabolic and cardiovascular complications.

Identify pharmaceutical compounds that improve mitochondrial metabolism in humans.

Determine metabolomic and lipidomic footprints of metabolic flexibility.

## Abbreviations

Acetylation: reversible protein modification, often on lysine residues, that can alter protein activity
Caloric restriction: prolonged reduction in caloric intake, typically 20-50%, while maintaining vitamin and mineral levels
Intramyocellular lipids: lipids that accumulate within the muscle. Associated with lipotoxicity
Metabolic flexibility: the ability of a cell system to efficiently switch between different nutrient sources
Mitochondrial dynamics: mitochondria display highly dynamic activity, consisting of continue cycles of fusion and fission
Mitochondrial fission: division of mitochondria during which daughter mitochondria are subjected to quality control
Mitochondrial fusion: process during which content of separate mitochondria is mixed
Mitophagy: specific autophagy for mitochondria; breakdown of malfunctioning mitochondria associated with quality control
NAD^+^: redox metabolite. Its role in regulating metabolism was emphasized after the identification of the NAD^+^-dependent sirtuin proteins
